# Plastid Genome Evolution in the Subtribe Calypsoinae (Epidendroideae, Orchidaceae)

**DOI:** 10.1093/gbe/evaa091

**Published:** 2020-05-14

**Authors:** Zhang-Hai Li, Yan Jiang, Xiao Ma, Jian-Wu Li, Jun-Bo Yang, Jian-Yong Wu, Xiao-Hua Jin

**Affiliations:** e1 State Key Laboratory of Systematic and Evolutionary Botany, Institute of Botany, Chinese Academy of Sciences, Beijing, China; e2 University of Chinese Academy of Sciences, Beijing, China; e3 Xishuangbanna Tropical Botanical Garden, Chinese Academy of Sciences, Yunnan, China; e4 Germplasm Bank of Wild Species in Southwest China, Yunnan, China; e5 Ministry of Ecology and Environment (MEE), Nanjing Institute of Environmental Sciences, Jiangsu, China

**Keywords:** *Corallorhiza*, Calypsoinae, *Danxiaorchis*, plastome, *Risleya*, mycoheterotrophy

## Abstract

Calypsoinae is a small subtribe in Orchidaceae (Epidendroideae) characterized by diverse trophic strategies and morphological characters. Calypsoinae includes 13 genera, four of which are leafless and mycoheterotrophic. Mycoheterotrophic species in the leafless genus *Corallorhiza* are well suited to studies of plastome evolution. However, the lack of plastome sequences for other genera in Calypsoinae limits the scope of comparative and phylogenetic analyses, in particular our understanding of plastome evolution. To understand plastid genome evolution in Calypsoinae, we newly sequenced the plastomes of 12 species in the subtribe, including representatives of three mycoheterotrophic genera as well as five autotrophic genera. We detected two parallel photosynthetic losses in *Corallorhiza*. Evolutionary analyses indicated that the transition to obligate mycoheterotrophy leads to the relaxation of selection in a highly gene-specific pattern.

## Introduction

Mycoheterotrophic species have received substantial attention owing to their unique lifestyle (Delannoy et al. 2011; Schelkunov et al. 2015; Barrett et al. 2018; Tsukaya 2018; Kim et al. 2019), in which they obtain carbon from fungi instead of photosynthesis (Merckx, Freudenstein, et al. 2013). At least, 47 independent origins of full mycoheterotrophy in land plants have been reported (Merckx, Mennes, et al. 2013).

There were about 25 independent origins of mycoheterotrophy in the family Orchidaceae (Merckx, Mennes, et al. 2013). The subtribe Calypsoinae (Epidendreae, Epidendroideae) includes 13 genera, four of which are mycoheterotrophic (Chase et al. 2015; Freudenstein et al. 2017). In Calypsoinae, the leafless genus *Corallorhiza* has been established as an ideal system for studies of plastome evolution in heterotrophic lineages (Barrett and Davis 2012; Barrett et al. 2014, 2018). However, the plastomes of Calypsoinae species published to date are limited to the genus *Corallorhiza*, emphasizing the need for sequence data for other genera, especially closely related autotrophic clades.

To characterize plastome evolution across Calypsoinae, we newly sequenced 12 Calypsoinae plastomes, including representative species of three mycoheterotrophic genera (*Corallorhiza*, *Danxiaorchis*, and *Risleya*) and five autotrophic genera.

## Materials and Methods

### DNA Extraction and Sequencing

Twelve species from eight Calypsoinae genera were sampled, including *Calypso bulbosa*, *Changnienia amoena*, *Corallorhiza trifida*, *Cremastra appendiculata*, *Danxiaorchis singchiana*, and *Risleya atropurpurea*, four *Oreorchis* species, and two *Tipularia* species (supplementary table S1, Supplementary Material online). *Agrostophyllum callosum* (Agrostophyllinae) was sampled as an autotrophic outgroup for comparative analyses. Fourteen previously published plastomes obtained from the NCBI database were included in the analyses (supplementary table S1, Supplementary Material online).

Total genomic DNA was extracted from silica-dried materials using a modified cetyltrimethylammonium bromide (CTAB) method (Li et al. 2013). DNA with a concentration of >100 ng/ml was sheared to fragments of ∼400–600 bp using Covaris M220. The NEBNext Ultra DNA Library Prep Kit (New England Biolabs, USA) was used to prepare DNA libraries for sequencing according to the manufacturer’s protocol. Paired-end sequencing (100- or 150-bp read lengths) was performed on the Illumina HiSeq 2500 platform at the Institute of Botany, Chinese Academy of Sciences, which generated ∼10 GB of raw data for heterotrophic species and at least 2 GB for autotrophic species.

### Plastome Assembly and Annotation

Plastome assembly was performed following previously described methods (Feng et al. 2016). In brief, raw reads were trimmed and filtered using NGSQCTOOLKIT v. 2.3.3 (Patel and Jain 2012), and bases with PHRED quality scores of lower than 20 were trimmed. The filtered paired reads were mapped to the plastome of *Calanthe triplicata* (NC_024544.1) using Geneious v. 10.1.2 to filter reads matching the reference genome. De novo assemblies were constructed using VELVET (Zerbino and Birney 2008) with K-mer values from 37 to 45. Contigs from consensus sequences were merged and combined into scaffolds using Geneious with default parameters. Scaffolds were extended by mapping reads using Geneious. The assembly steps were repeated to obtain the draft plastome. Assembly errors were corrected by mapped all sequencing reads to draft plastome. IR boundaries for each plastome were confirmed by BLAST and the reverse IR was added to complete plastome by hand.

Completed plastomes were annotated using PGA (Qu et al. 2019) with the annotated plastome of *Calanthe triplicata* as a reference, followed by manual checking and adjustment of gene or exon boundaries using Geneious v. 10.1.2. The initiation codon, termination codon, and other annotation errors for each gene were revised using Sequin, and results were exported as GenBank files.

### Phylogenetic Analysis

To reconstruct phylogenetic relationships, all protein-coding sequences (supplementary table S4, Supplementary Material online) were exported from plastomes using Geneious v. 10.1.2. Each single gene matrix was aligned using MAFFT under the automatic model selection option (Katoh and Standley 2013) with manual adjustments in BioEdit (Hall 1999). These were subsequently combined into a single plastome supermatrix using PhyloSuite (Zhang et al. 2020). The concatenated sequences were used for a phylogenetic analysis by the maximum likelihood (ML) method using IQ-TREE (Nguyen et al. 2015) with the best-fit model TVM + F + R3 was automatically selected by ModelFinder (Kalyaanamoorthy et al. 2017). Branch support was evaluated by 1,000 bootstrap replicates (-bb 1000).

### Molecular Evolutionary Analyses

Plastomes of mycoheterotrophic Calypsoinae are highly reduced (see Results and Discussion), there are 25 common protein-coding genes (CDS) in all sampled plastomes (see table 1 and supplementary table S4, Supplementary Material online). These CDS were aligned at the codon level with the option “-codon” using MUSCLE in MEGA v. 7.0.2 (Kumar et al. 2016). Stop codons were removed from the sequences prior to alignment. The output topology from the ML phylogenetic analysis based on all of the protein-coding sequences was used for the evolutionary analysis. CODEML in the PAML software package (Yang 2007) was used to calculate branch lengths of d*S* and d*N* of the 25 concatenated protein-coding genes.

**Table 1:** The Number of Genes, Length, and GC Content of the Newly Sequenced Plastid Genomes in This Study

Species	Number of Genes			Length (bp)				GC Content (%)			
	Protein Coding	tRNA	rRNA	Total	LSC	IR	SSC	Total	LSC	IR	SSC
*Agrostophyllum callosum*	79	30	4	158,365	86,349	26,743	18,530	37.0	34.8	43.2	29.9
*Calypso bulbosa*	69	30	4	149,451	83,800	25,654	14,343	37.1	34.5	43.5	29.1
*Changnienia amoena*	79	30	4	156,828	84,856	26,915	18,142	37.1	34.8	43.2	29.8
*Corallorhiza trifida_*14268	68	30	4	149,408	83,171	25,920	14,397	37.2	34.5	43.7	28.8
*Cremastra appendiculata*	79	30	4	159,493	86,948	27,089	18,367	37.0	34.6	43.3	29.9
*Danxiaorchis singchiana*	29	22	4	87,910	42,494	13,763	17,890	34.6	31.1	37.0	39.0
*Oreorchis angusta*	79	30	4	158,654	86,294	27,024	18,312	37.0	34.6	43.2	29.8
*Oreorchis foliosa*	75	30	4	158,496	86,061	27,037	18,361	36.9	34.6	43.2	29.6
*Oreorchis indica*	75	30	4	158,599	86,109	27,437	17,616	36.9	34.6	43	29.6
*Oreorchis patens*	79	30	4	158,256	86,253	26,831	18,341	37.0	34.6	43.3	29.6
*Risleya atropurpurea*	25	15	4	77,821	20,656	26,260	4,645	33.3	25.7	37.5	19.2
*Tipularia josephii*	68	30	4	146,816	82,881	25,977	11,981	37.4	34.9	43.5	28.2
*Tipularia szechuanica*	68	30	4	142,799	82,378	24,444	11,533	37.4	34.9	43.6	28.7

The selection intensity parameter (*k*) was calculated using RELAX (Wertheim et al. 2015) within the Datamonkey server (Delport et al. 2010). In particular, test branches (mycoheterotrophic species) under relaxed (*k* < 1) or intensified (*k* > 1) selection relative to the reference branches (autotrophic species) were examined. The significance of the *k* parameter was determined by likelihood ratio tests.

## Results and Discussion

### Phylogenetic Relationships and Photosynthetic Losses of Calypsoinae

Plastome data provided better understanding of phylogenetic relationships and losses of photosynthesis within Calypsoinae (supplementary fig. S1, Supplementary Material online). Our phylogenetic analysis of interrelationships of Calypsoinae is generally consistent with previous studies (Chase et al. 2015; Freudenstein et al. 2017; Li et al. 2019) but provides new insight into relationships among taxa. *Corallorhiza* was not monophyletic, including two groups nested within *Oreorchis* lineages. *Danxiaorchis* and *Cremastra* were sister groups. We detected four independent losses of photosynthesis in Calypsoinae, two of which were parallel losses in *Corallorhiza*. Parallel photosynthetic losses have been observed in *Hexalectris* (Barrett et al. 2019), and our results indicated that parallel photosynthetic losses may be more common in Orchidaceae than expected.

### Genome Size, Gene Content, and Structure of Calypsoinae Plastids

**Fig. 1.**
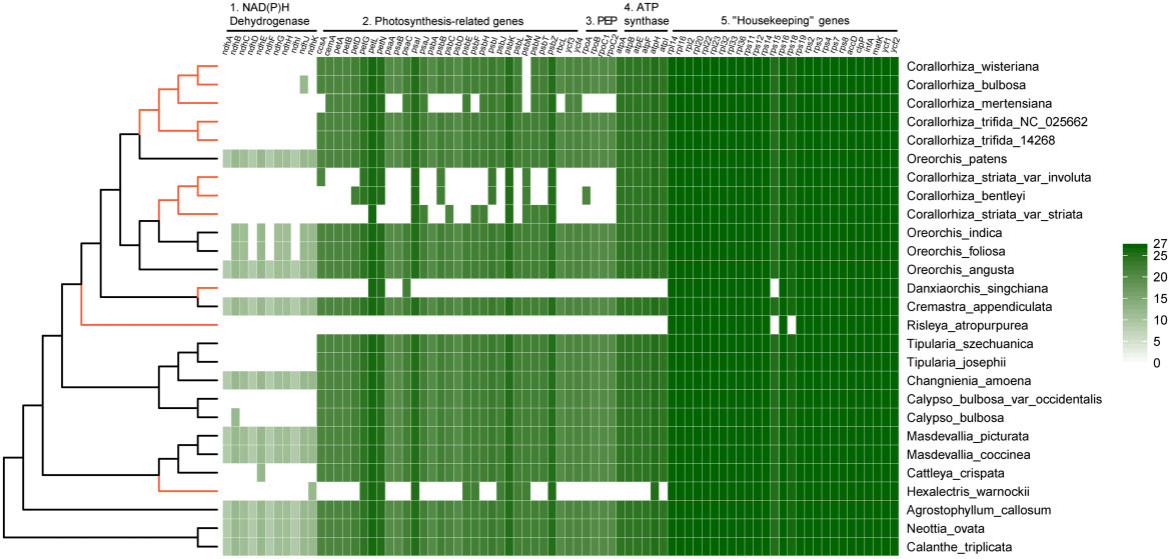
—Summary of retained genes in the plastomes of sampled species. Intact genes per species are indicated by green boxes, whereas the white boxes mark functional and or physical losses. The shading boxes (0–27) indicate the number of species in this study retaining the genes. Orange branch indicates leafless and mycoheterotrophic species.

Plastid genomes of sampled Calypsoinae species ranged in size from 77,821 bp in *R. atropurpurea* to 159,493 bp in *Cremastra appendiculata* (table 1). In mycoheterotrophic species, we observed high variation in genome size, from 77,821 to 149,384 bp. The fully mycoheterotrophic *R. atropurpurea* plastome (77,821 bp) was the most highly reduced among the plastomes of Calypsoinae species sequenced to date (table 1). The plastid genome of the mycoheterotrophic species *D. singchiana* also showed a dramatic reduction to 87,910 bp (table 1). The loss or pseudogenization of *rps* genes, such as *rps*15 in *D. singchiana* and *rps*15 and *rps*18 in *R. atropurpurea* (fig. 1), is consistent with the final stage of the plastome degradation model proposed by Barrett and Davis (2012).

The highly reduced plastomes of *R. atropurpurea* and *D. singchiana* still had a quadripartite structure but exhibited a shift in the IR/SC region boundary. The plastome of *R. atropurpurea* was basically collinear with those of autotrophic relatives (supplementary fig. S2, Supplementary Material online), but *rpl*36, *inf*A, *rps*8, *rpl*14, *rpl*16, and *rps*3, which are usually present in the large single copy region, were translocated to the IR region. In plastome of *D. singchiana*, all *rrn* genes were translocated from the IR region to the SC region. In addition, we detected two inversions in the plastome of *D. singchian*a (supplementary fig. S2, Supplementary Material online).

### Rate of Evolution and Selection

Mycoheterotrophic species exhibited a higher substitution rate and thereby longer branches relative to those of autotrophic lineages (supplementary fig. S3, Supplementary Material online). Both synonymous and nonsynonymous substitution rates of *R. atropurpurea* were higher than those of other Calypsoinae species (supplementary fig. S3, Supplementary Material online). The RELAX model predicted that the relative strength of selection (relaxation or intensification) varied among plastid protein-coding genes in mycoheterotrophic species. In particular, 15 of 25 common genes displayed evidence of relaxed selective constraint associated with the loss of photosynthesis, including a significant relaxation of selective constraint on five genes, *acc*D, *rpl*23, *rps*11, *rps*16, and *ycf*1 (*P *<* *0.05; supplementary table S2, Supplementary Material online). Analyses of concatenated gene sets for each functional class revealed evidence for significantly relaxed selection in ACIM (*acc*D, *clp*P, *inf*A, and *mat*K) and the *rps* complex (*P *<* *0.05; supplementary table S3, Supplementary Material online).

Based on previous molecular dating, the estimated crown age of *Corallorhiza* is ∼9 Myr (Barrett et al. 2018), whereas *Danxiaorchis* split from its autotrophic sister group 11–12 Ma (Barrett et al. 2018; Li et al. 2019) and *Risleya* diverged about 24 Ma (Li et al. 2019). Based on these estimates and the degree of plastome reduction in the three lineages (fig. 1), the time since the shift to mycoheterotrophy might be the main determinant of the degree of plastome reduction and elevated evolutionary rate in heterotrophic lineages (Barrett et al. 2019).

## Supplementary Material

evaa091_Supplementary_Data

## Notes

**Data deposition:** This project has been deposited at NCBI under the accessions MN990431–MN990443.
