# Supplementary material for: Plastid Genome Evolution in the Subtribe Calypsoinae (Epidendroideae, Orchidaceae)
Source: Genome Biol Evol. 2020 May 14;12(6):867–70. doi: 10.1093/gbe/evaa091 (PMC7313661; doi:10.1093/gbe/evaa091)
Supplement: evaa091_Supplementary_Data [file evaa091_supplementary_data.zip › Table S1..docx]

**Table S1. Voucher and NCBI accession number of plastomes of sampling used in this study.** (Voucher is specimen, information including collector, collection number and herbarium. PE, Herbarium, Institute of Botany, Chinese Academy of Sciences; KUN, Herbarium, Kunming Institute of Botany, Chinese Academy of Sciences)

| Species | NCBI accession | Voucher |
| --- | --- | --- |
| *Agrostophyllum callosum* | MN990432, newly sequenced | Xiaohua Jin 13044 (PE) |
| *Calypso bulbosa* | MN990433, newly sequenced | Zhou HC 6237(KUN) |
| *C. bulbosa* var. *occidentalis* | MG874037 |  |
| *Cattleya crispata* | NC_026568 |  |
| *Changnienia amoena* | MN990431, newly sequenced | Xiaohua Jin 21024 (PE) |
| *Corallorhiza bentleyi* | MG874035 |  |
| *C. bulbosa* | NC_025659 |  |
| *C. mertensiana* | NC_025661 |  |
| *C. striata* var*. involuta* | MG874038 |  |
| *C. striata* var. *striata* | MG874034 |  |
| *C. trifida* | NC_025662 |  |
| *C. trifida_*14268 | MN990435, newly sequenced | Xiaohua Jin 14268 (PE) |
| *C. wisteriana* | NC_025663 |  |
| *Cremastra appendiculata* | MN990434, newly sequenced | Xiaohua Jin 10357 (PE) |
| *Danxiaorchis singchiana* | MN990438, newly sequenced | Xiaohua Jin 17000 (PE) |
| *Hexalectris warnockii* | MH444822 |  |
| *Masdevallia coccinea* | NC_026541 |  |
| *M. picturata* | NC_026777 |  |
| *Oreorchis angusta* | MN990443, newly sequenced | Xiaohua Jin 7897 (PE) |
| *O. foliosa* | MN990441, newly sequenced | Xiaohua Jin 13183 (PE) |
| *O. indica* | MN990440, newly sequenced | Xiaohua Jin 20498 (PE) |
| *O. patens* | MN990436, newly sequenced | Xiaohua Jin 20304 (PE) |
| *Risleya atropurpurea* | MN990439, newly sequenced | Xiaohua Jin 20214 (PE) |
| *Tipularia josephii* | MN990437, newly sequenced | Xiaohua Jin 13301 (PE) |
| *T. szechuanica* | MN990442, newly sequenced | Jin et al. ST-2248 (PE) |
| Outgroup |  |  |
| *Calanthe triplicata* (Collabieae) | NC_024544 |  |
| *Neottia ovata*  (Neottieae) | NC_030712 |  |
