# Supplementary material for: Plastid Genome Evolution in the Subtribe Calypsoinae (Epidendroideae, Orchidaceae)
Source: Genome Biol Evol. 2020 May 14;12(6):867–70. doi: 10.1093/gbe/evaa091 (PMC7313661; doi:10.1093/gbe/evaa091)
Supplement: evaa091_Supplementary_Data [file evaa091_supplementary_data.zip › Table S3.docx]

**Table S3.** **Results of RELAX analyses based on genes concatenated by functional class.**

| Gene | Model | logL | No. of params | AICc | Branch | ω1 | ω2 | ω3 | K | P-value | LR | Selection |
| --- | --- | --- | --- | --- | --- | --- | --- | --- | --- | --- | --- | --- |
| ACIM* | Alternative | -14260.3 | 76 | 28672.9 | Reference | 0.00 (43.11%) | 0.78 (56.42%) | 89.60 (0.47%) | 0.77 | 0.003 | 9 | **relaxation** |
|  |  |  |  |  | Test | 0.00 (43.11%) | 0.82 (56.42%) | 31.27 (0.47%) |  |  |  |  |
|  | Null | -14264.8 | 75 | 28679.9 | Reference | 0.00 (44.06%) | 0.84 (55.56%) | 67.56 (0.39%) |  |  |  |  |
|  |  |  |  |  | Test | 0.00 (44.06%) | 0.84 (55.56%) | 67.56 (0.39%) |  |  |  |  |
| ycf** | Alternative | -43504.5 | 72 | 87153 | Reference | 0.00 (0.32%) | 0.85 (99.53%) | 249.92 (0.15%) | 1.24 | 0.230 | 1.44 | intensification |
|  |  |  |  |  | Test | 0.00 (0.32%) | 0.81 (99.53%) | 964.34 (0.15%) |  |  |  |  |
|  | Null | -43505.2 | 71 | 87152.5 | Reference | 0.00 (0.33%) | 0.84 (99.48%) | 240.58 (0.19%) |  |  |  |  |
|  |  |  |  |  | Test | 0.00 (0.33%) | 0.84 (99.48%) | 240.58 (0.19%) |  |  |  |  |
| rpl | Alternative | -8490.9 | 76 | 17134.1 | Reference | 0.00 (62.61%) | 1.00 (37.31%) | 130.64 (0.08%) | 4.86 | 0.059 | 3.55 | intensification |
|  |  |  |  |  | Test | 0.00 (62.61%) | 1.00 (37.31%) | 19268228516.35 (0.08%) |  |  |  |  |
|  | Null | -8492.6 | 75 | 17135.7 | Reference | 0.02 (64.06%) | 1.00 (35.88%) | 2946.57 (0.05%) |  |  |  |  |
|  |  |  |  |  | Test | 0.02 (64.06%) | 1.00 (35.88%) | 2946.57 (0.05%) |  |  |  |  |
| rps | Alternative | -12974.5 | 76 | 26101.3 | Reference | 0.22 (5.12%) | 0.23 (94.63%) | 127.91 (0.25%) | 0.70 | 0.000 | 13.26 | **relaxation** |
|  |  |  |  |  | Test | 0.35 (5.12%) | 0.36 (94.63%) | 29.88 (0.25%) |  |  |  |  |
|  | Null | -12981.2 | 75 | 26112.6 | Reference | 0.23 (85.01%) | 1.00 (14.89%) | 339.80 (0.09%) |  |  |  |  |
|  |  |  |  |  | Test | 0.23 (85.01%) | 1.00 (14.89%) | 339.80 (0.09%) |  |  |  |  |

*ACIM: *acc*D, *clp*P, *inf*A, *mat*K

** ycf: *ycf*1, *ycf*2
