# Supplementary figures and images for: Plastid Genome Evolution in the Subtribe Calypsoinae (Epidendroideae, Orchidaceae)
Source: Genome Biol Evol. 2020 May 14;12(6):867–70. doi: 10.1093/gbe/evaa091 (PMC7313661; doi:10.1093/gbe/evaa091)

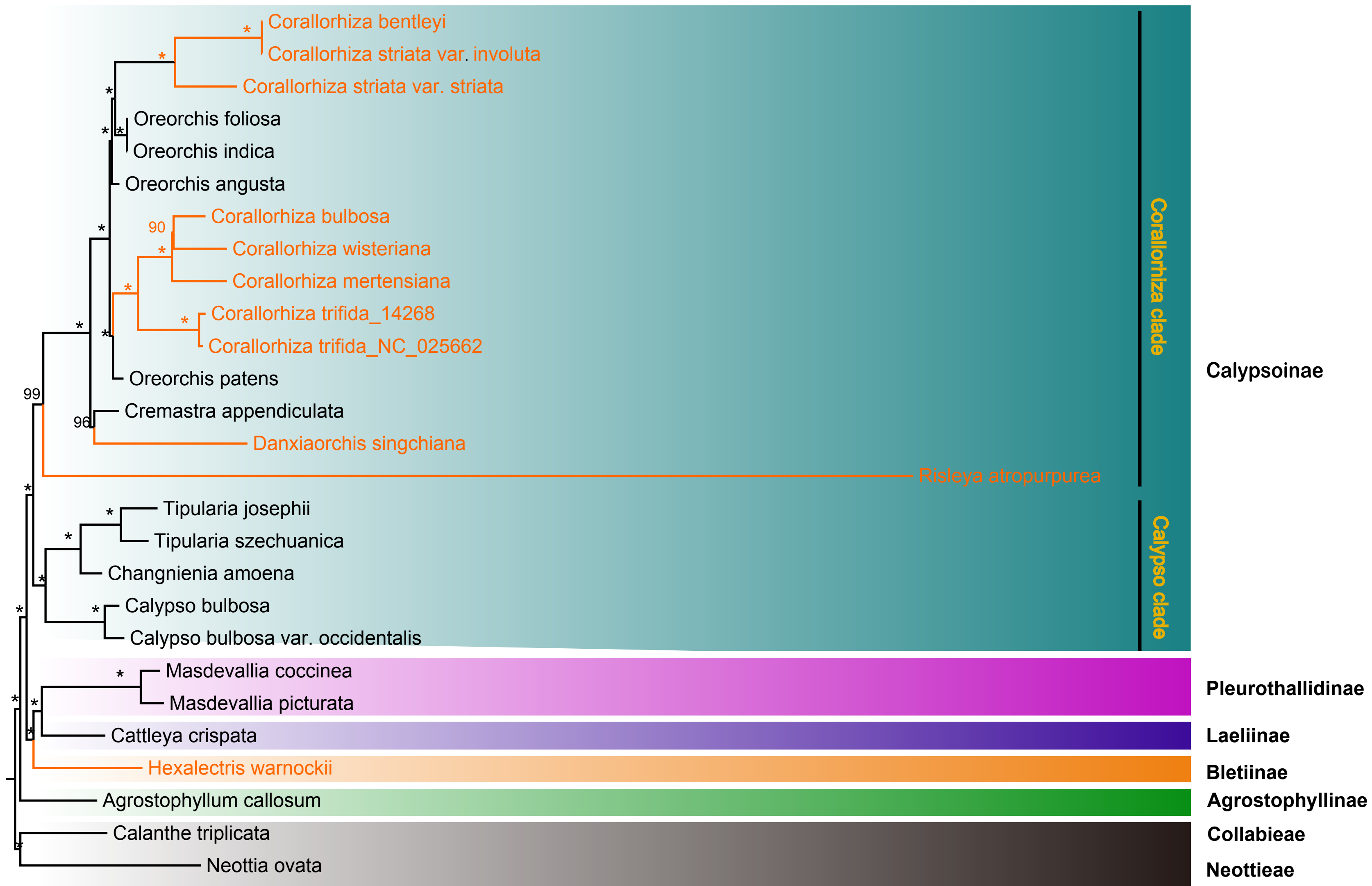

Supplement: evaa091_Supplementary_Data [file evaa091_supplementary_data.zip › Figure S1. Phylogram of Calypsoinae.pdf]

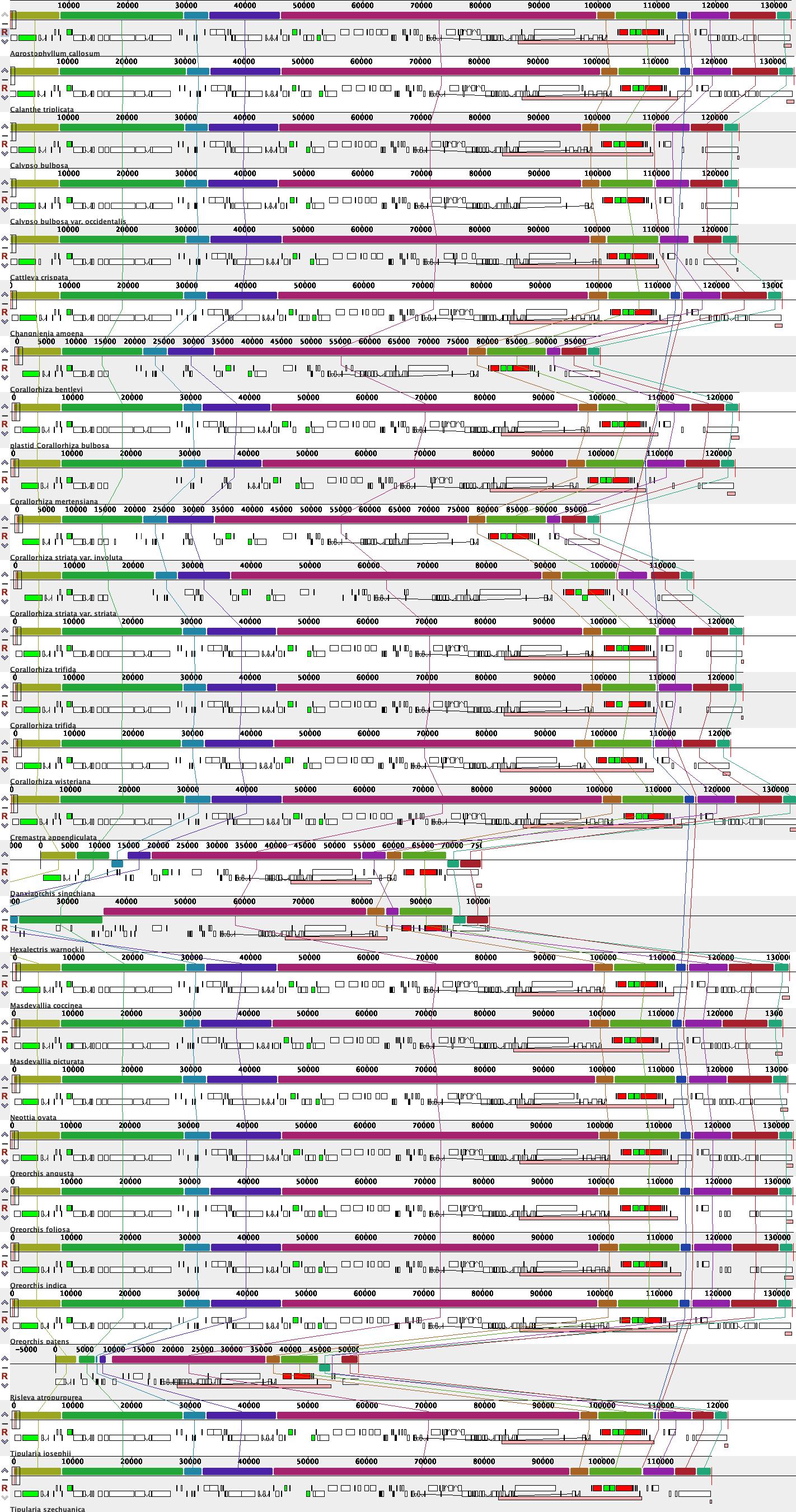

Supplement: evaa091_Supplementary_Data [file evaa091_supplementary_data.zip › Figure S2.jpeg]

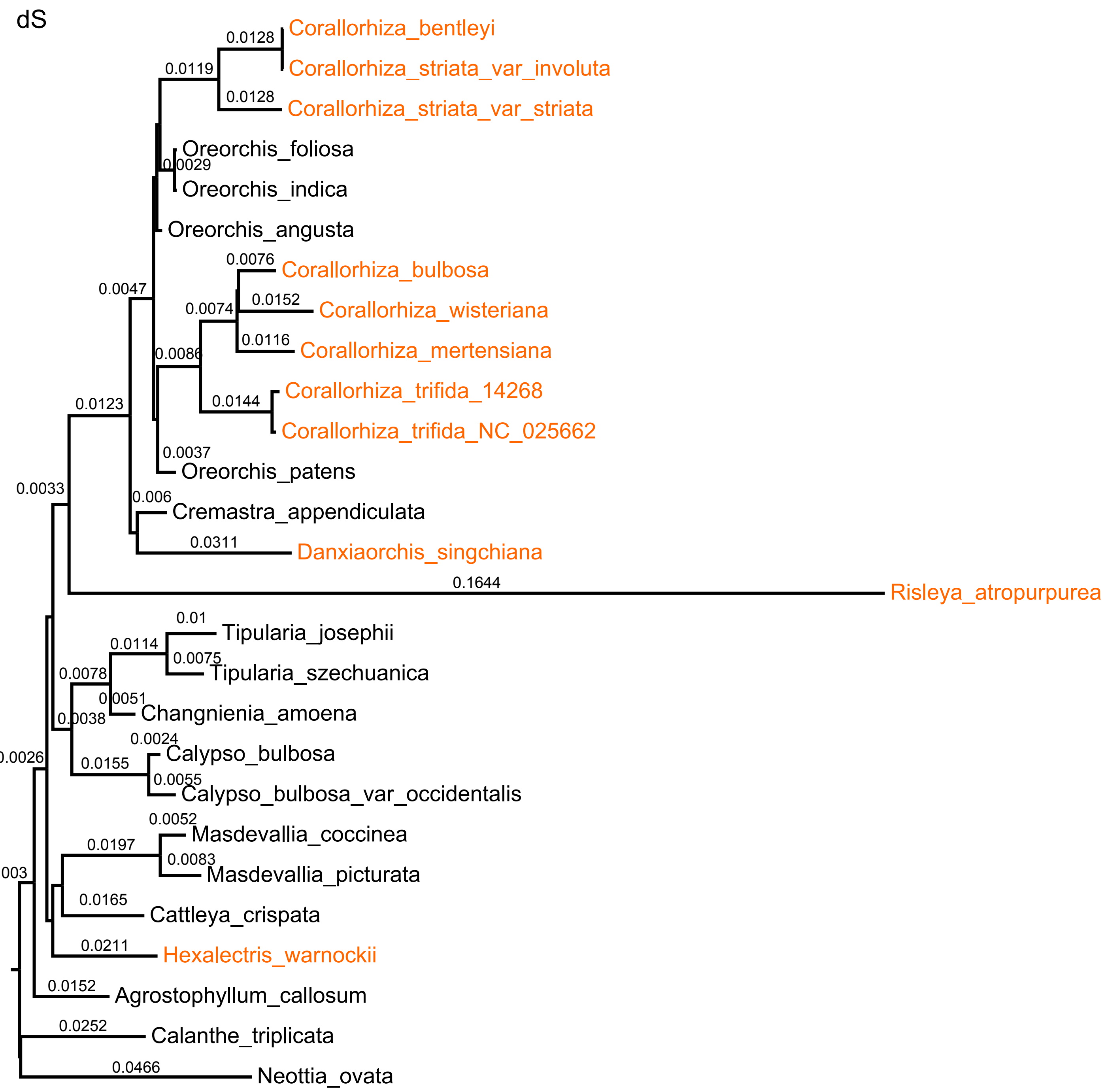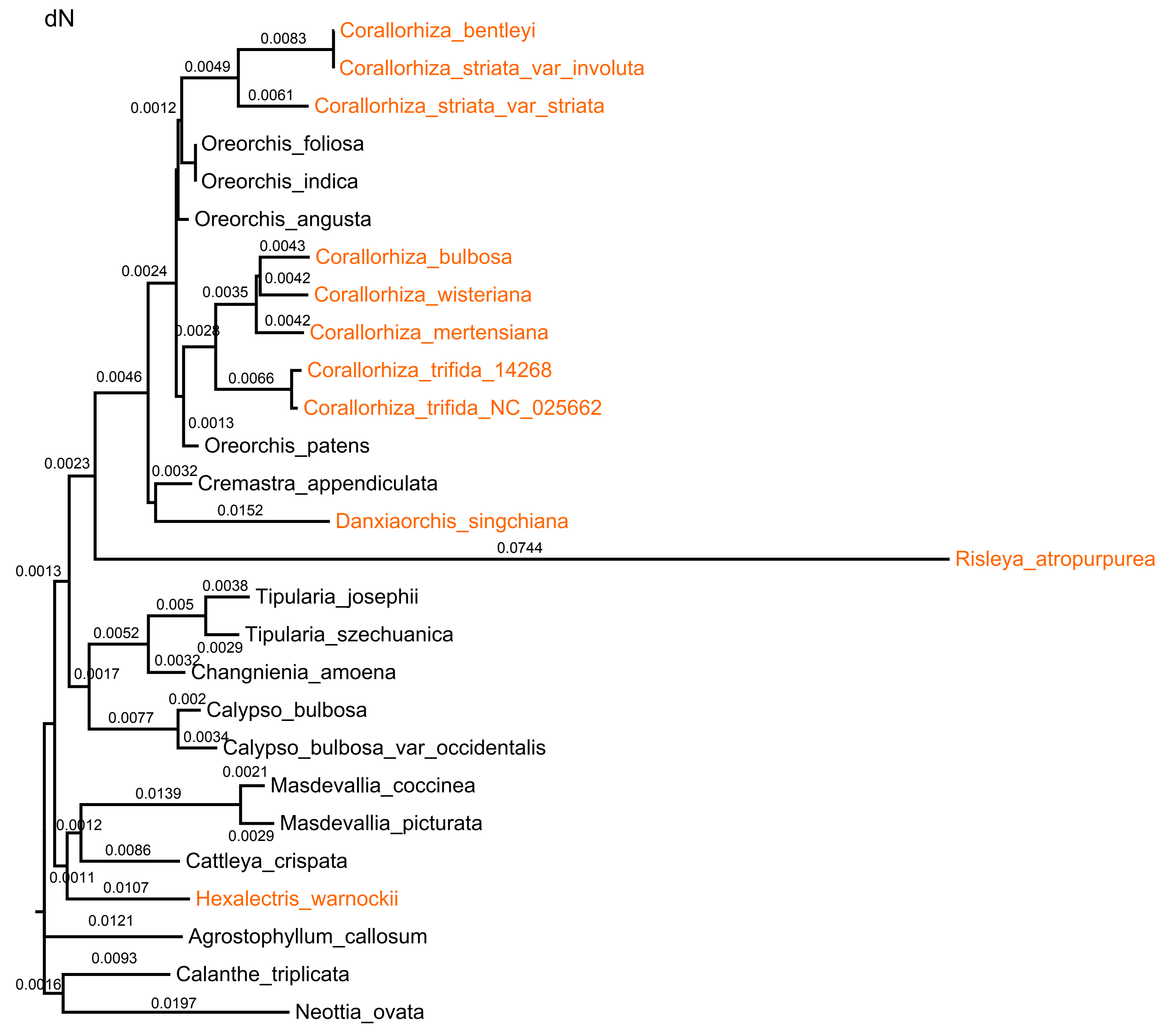

0.02

0.008

Supplement: evaa091_Supplementary_Data [file evaa091_supplementary_data.zip › Figure S3.pdf]
